# Ocular Complications in Cutaneous Lupus Erythematosus: A Systematic Review with a Meta-Analysis of Reported Cases

**DOI:** 10.1155/2015/254260

**Published:** 2015-06-11

**Authors:** L. Arrico, A. Abbouda, I. Abicca, R. Malagola

**Affiliations:** Department of Ophthalmology, Sapienza University, Umberto I Hospital, Viale del Policlinico 155, 00186 Rome, Italy

## Abstract

Ocular complications associated with cutaneous lupus erythematosus (CLE) are less studied compared with those ones associated with systemic lupus erythematosus (SLE). The main ocular sites involved in patients affected by discoid lupus erythematosus (DLE) are eyelids followed by orbit and periorbit, the least being cornea. The most common complications are blepharitis usually affecting the lower lid and associated with some type of lid lesion such as plaque or erythematosus patches and madarosis. Few cases with LE profundus (LEP) and ocular complications are reported, but they are associated with orbital inflammatory syndrome and severe complications. The main treatment prescribed is hydroxychloroquine with a dose of 200 mg twice a day for 6 to 8 weeks. Corticosteroids are also used. Intervals between the correct diagnosis and the beginning of the ocular symptoms are commonly delayed. Ophthalmologist should be aware of the ocular manifestation of this autoimmune disease.

## 1. Introduction

Cutaneous lupus erythematosus (CLE) encompasses a wide range of dermatologic manifestations and is two to three times more frequent than systemic lupus erythematosus (SLE) [1, 2].

Cutaneous lupus is divided into several subtypes, including acute CLE (ACLE), subacute CLE (SCLE), and chronic CLE (CCLE). CCLE includes discoid lupus erythematosus (DLE), LE profundus (LEP), chilblain LE (CHLE), and LE tumidus (LET) [3–5].

Discoid lesions are the most common form of CCLE [1–6]. DLE is more frequent in women during their fourth and fifth decade of life [7]. 60–80% of discoid lesions are localized in sun exposed areas, such as head, neck, the scalp, ears, and cheeks [8–12]. Occasionally, DLE can occur on mucosal surfaces, including lips and oral, nasal, and genital mucosa [1].

Cutaneous lesions begin as erythematosus maculae or papules with a scaly surface, which gradually grow peripherally into larger adherent discoid plaques that heal leaving an atrophic scar and pigmentary changes [1–6].

Histological examination of a longstanding active DLE lesion reveals hyperkeratosis, dilated compact keratin-filled follicles, vacuolar degeneration of the basal keratinocytes, and an intensely inflammatory dermal infiltrate.

Serologically, DLE patients have a lower incidence of ANA, dsDNA, Sm, U1RNP, and Ro/SSA antibodies, compared to other CLE subtypes [13]. Ninety percent of DLE lesions have a positive lupus band test with C3 and IgM as the most common immune deposits [14].

Less common form of CLE is LEP. This is a painful panniculitis with subcutaneous nodules in the lower dermis and subcutaneous adipose tissue. The most common area involvements are the upper arms and legs, face, and breasts. Histology shows lobular panniculitis with a dense lymphocytic infiltrate [15]. LEP tends to a chronic course, leaving atrophic scars [16].

Ocular complications in CLE are not so represented and few cases are reported [17–56]. The aim of this paper is to analyze the ocular association with this autoimmune disease and offer a systematic view of the treatment regarding the ocular involvement.

## 2. Materials and Methods

Published journal articles are considered as the elements of study and a specific literature search is performed in four stages.

**Stage 1 (unique citations).**

A Medline (National Library of Medicine, Bethesda, Maryland, USA) search from January 1983 to December 2014 is performed to identify all articles describing ocular complications in patients with DLE. Keyword searches used included “discoid lupus” + “ocular complications” and “cutaneous lupus” and “ocular.”

**Stage 2 (article retrieval).**

All abstracts from the Medline searches are scrutinized to identify articles that reported clinical results. Only journal articles published in English are included. Copies of the articles are obtained, and their bibliographies are searched manually for additional articles published in peer-reviewed journals.

**Stage 3 (article inclusion).**

Complete articles are reviewed to identify those that reported original clinical data or complication(s) of CLE. We decide to include 41 articles.

**Stage 4 (article exclusion).**

We exclude all articles that describe diagnosis different from CLE, published in a different language from English and where it is not possible to obtain the full text form. We also avoid including articles where the details regarding the disease or the treatment prescribed for each patient reported are not available.

## 3. Results

A total of 41 articles are selected [17–56]. Tables 1 and 2 summarize data items from each paper. All papers are case reports or case series. The total number of patients with CLE and ocular complications is seventy-seven (111 eyes). Sixteen subjects (20.8%) are male and sixty-one (79.2%) female. The average age is 43.37 ± 15 years (range: 17–89 years). The most represented ethnicity is Caucasian (25 cases; 32.5%) followed by African (9 cases; 11.7%), Asian (6 cases; 7.8%), Caribbean (4 cases; 5.2%), and Indian (1 case; 1.3%). In thirty-two cases (41.6%), the ethnicity is not specified. Seventy-one patients have a diagnosis of DLE (92.2%) and six patients of LEP (7.8%). Interval between the correct diagnosis and the beginning of the symptoms is an average of 41.40 ± 54.9 months in the group of patients with DLE, while in patients with LEP it is shorter (an average of 1.8 ± 2.4 months). This difference is statistically significant (*P* = 0.02).

Among patients with DLE, the ocular complications are monolateral in 38 cases (53.5%) and bilateral in 33 cases (46.5%). The main ocular site involved in these patients is the eyelids. This site is affected in 63 patients (88.7%) followed by orbit and periorbit in six cases (8.4%) and cornea in 2 cases (2.6%).

Extraocular involvement is commonly reported. Facial lesions are described in 66 cases (93%). The most frequent facial lesions are localized on forehead philtrum, malar region, nose, and cheek and perioral nostril rim was less common. Body lesions are found in four patients (5.6%). The anatomic sites are arm back and knee. Alopecia is also a finding represented in three cases (4.2%).

Blepharitis is the most represented ocular complication. It affects thirty-eight patients (53.5%). The lower eyelid is the most common site (27 cases; 38%), upper and lower lids together (4 cases; 5.6%), and upper lid (1 case; 1.4%). In thirty-nine cases (54.9%), this data is not reported. Telangiectasia over the lid is described in two cases (2.8%).

Madarosis is associated with twenty cases (28.2%). In sixteen cases (22.5%) lid lesions, such as lid plaques, are described. In eight cases (11.3%) erythematosus papulae or maculae close to the eyelid are described. Lid edema or swelling is reported in nine cases (12.7%). Anomalies in lid pigmentation are reported in three cases (4.2%). One case of area of depigmentation and two cases of hyperpigmented lesions are also reported.

Cornea involvement is described in two cases (5.6%) such as stromal keratitis, but also in another two patients, a punctate keratopathy is described associated with blepharitis.

ANA resulted to be negative in 21 cases (29.6%) and positive in 15 cases (21.1%). The pattern most represented is a speckled pattern (6 cases; 8.5%) followed by nucleolar pattern (3 cases; 4.2%) and diffuse pattern (1 case; 1.4%). In five cases (7%), the pattern is not specified.

The six patients (7.8%) with a diagnosis of LEP associated with ocular complications show some peculiar features. Monolateral form is the most representative (five cases; 85.7%). The ocular site more involved is orbit and periorbit (five cases; 83.3%). In the other case, the ocular complication involved eyelids. Facial skin lesions are described in almost all cases (5 cases; 85.75%).

The treatment prescribed after the diagnosis of DLE is hydroxychloroquine (40 cases; 51.9%).

The most common dosage prescribed is 200 mg twice a day. The weeks of treatment performed are reported in only six cases. The average time of treatment is 28.8 ± 36.9 weeks (range: 2–96).

Some cases of allergies to hydroxychloroquine are reported.

The second choice of treatment is corticosteroid. This is the first choice in all cases affected by LEP. Intravenous methylprednisolone (1 g/day) or oral prednisolone with a dosage between 20 mg/day and 60 mg/day is the most common choice. Some cases are treated by topical corticosteroids drops, corticosteroid ointment, or tacrolimus ointment. In two cases where the hydroxychloroquine is not prescribed, the drug selected is mycophenolate. CO_2_ laser is used to remove some lid lesions.

A good control of the disease is obtained in the majority of the patients and the time of follow-up reported is 4 ± 11.7 months (range: 0–82). Important complications related to orbital inflammatory syndrome such as central retinal artery occlusion and enophthalmos are described in the group with LEP.

## 4. Discussion

CLE is two to three times more frequent than SLE [2] but ocular complications are less represented compared to SLE.

Ocular involvement in patients with CLE is not so common. Among CLE subtypes, cases with DLE and ocular complications are mostly described comparing cases affected by LEP. In 1930, Aubaret [57] presented the first case of chronic blepharitis in patients with DLE and, after 30 years, Duke-Elder [58] and Donzi [59] reported additional cases. Since the early 80s, seventy-one cases of ocular complications associated with DLE are reported in the literature compared to only six cases of patients with LEP. No ocular complications are reported for the other CLE subgroups: ACLE, SCLE, CHLE, and LET.

In DLE, females are twice as likely to be affected as males. Most patients are between 20 and 60 years old, with a mean age of 42. Cutaneous manifestation of DLE may be slightly more common in African Americans than in Caucasians or Asians [14, 18, 60], but according to our data ocular complications associated with DLE are more common in Caucasian people. This data could be also biased because the probability that the medical cases are reported and described in the literature is higher in countries where the Caucasian ethnicity is most represented.

The localized form of DLE is characterized by the involvement of only the head and/or scalp and accounts for 70% of DLE patients. The generalized form has more extensive involvement and accounts for the remaining 30% of cases. Facial involvement is present in 74% of DLE patients, with the scalp and ear being the most commonly involved sites [16]. According to our data, 93% of patients have some facial lesions localized on forehead philtrum, malar region, nose, and cheek.

The peculiar locations of the DLE lesions in the perioral, periocular, and perinasal regions are described by Akagi et al. [31] and they are associated with a relatively good prognosis. This data is to be taken into account before prescribing the treatment. Actually, these patients improved without any treatment avoiding the risk of antimalarials drugs.

A biopsy of facial lesions and immunopathology is more useful to make a definitive diagnosis. The typical findings reported for DLE were the intense, thick granular IgG and IgM and complement C3 deposition along the basal membrane line [34].

Ocular manifestations of DLE reported in the literature include periorbital edema, blepharitis, madarosis, lid scarring, entropion and ectropion, trichiasis, panniculitis, conjunctivitis, hypertrophic/verrucous lesions, and stromal keratitis [17–56].

Blepharitis is the most common sign and in the majority of patients it is bilateral. In some cases the manifestation is asymmetric and in few cases it is monolateral.

Most lesions seem to occur on the inferior portion of the eyelid and are described as reddish, erythematosus, slightly infiltrated plaques, with or without scales, atrophy, or scarring.

Conjunctivitis, meibomitis, madarosis, and chronic eyelid erythema lid plaque lesions areas of anomaly pigmentation are reported. The differential diagnosis of DLE eyelid involvement includes rosacea blepharoconjunctivitis, seborrheic blepharitis, chronic staphylococcal blepharitis, contact dermatitis, eczema, psoriasis, sebaceous cell carcinoma, lid-involving sarcoidosis, lichen planus, lichenoid drug eruption, and tinea faciei [18, 19, 24, 60, 61].

Recent studies have identified histopathologic features of cutaneous lupus and this helped to distinguish it from squamous cell malignancies in extrapalpebral locations [62].

According to Papalas et al. [45], patients with CLE affecting the eyelid are older than patients with squamous cell carcinoma of the eyelid. In DLE the nodular/ulcerative eyelid lesions develop over a period of 6 months or less. A full-thickness eyelid biopsy is required when the diagnosis is doubtful, but it could lead to a poor cosmetic result and recurrent wound dehiscence [24].

An important cornea involvement is described in two cases [23]. The author reported an acute unilateral corneal stromal infiltration and edema responded to corticosteroid therapy without evidence of infection. He speculates that the cause of corneal involvement in this group of patients is related to a vasculitis near the limbus that may lead to inflammation and corneal edema.

In addition, cases with punctate keratopathy are described.

Orbital involvement is represented in the DLE patients, although it is not the main site involved. On the contrary, orbital involvement is the main site in patients with LEP. Eyelid complications in patients with LEP have been reported less frequently. Kearns et al. [63] reported a patient with LE profundus principally in the region of left zygomatic arch but extending to the lower eyelid and resulting in mild periorbital oedema. Nowinski et al. [64] described three patients with periorbital LEP, one of whom had proptosis and marked periorbital oedema. In the other two cases the eyelid oedema was less pronounced. Sheehan-Dare and Cunliffe [22] also reported a patient with marked periorbital oedema. This oedema was massive compared to common eyelid oedema described in DLE patients. However, proptosis and conjunctival involvement were not present while periorbital swelling and important proptosis were reported by Magee et al. [25].

Ohsie et al. [53] and Sudhakar et al. [54] described cases affected by LEP and orbital inflammatory syndrome. One of these cases [54] was complicated by a retinal artery occlusion probably due to compression of optic nerve by intraorbital fat. Kao et al. [55] reported a case of a man with diffuse red-violet discoloration of the right upper and lower eyelids with palpable induration and progressive enophthalmos.

The differential diagnosis of periorbital oedema is large. Important causes of unilateral oedema include orbital tumours that must be excluded. The presence of facial lesion associated with CLE has to be evaluated in the differential diagnosis. The percentage of males and females affected is more similar compared to DLE.

Only 20% of DLE patients have a positive ANA [60] and the most reported pattern among the patients with DLE and ocular complications is the speckled one.

The goal of therapy is to prevent the progression of existing lesions, improve patient appearance, and prevent further lesions. Sunglasses and sunscreens are recommended because ultraviolet exposure may exacerbate this disorder. In all patients the standard treatment for blepharitis, such as lid hygiene, doxycycline, and steroid creams, failed. The use of topical and intralesional corticosteroids may sometimes be helpful [21, 38, 45, 47], but the antimalarials are the mainstay of therapy. Hydroxychloroquine (200–400 mg orally per day) may take up to 6 to 8 weeks for a response and require monitoring every 6 months by ophthalmoscopy and visual fields to screen for medication-related maculopathy [14, 16, 65].

According to the data, the majority of patients responded to systemic hydroxychloroquine therapy and when antimalarials cannot be used or if the lesion is resistant, immunosuppressive can be considered. Agents that have been reported to be successfully used for DLE include azathioprine, dapsone, methotrexate, cyclophosphamide, thalidomide, retinoids, and interferon alpha-2 [14, 16, 27, 65].

Corticosteroids are mainly used in patients with LEP and orbital inflammatory syndrome to control the severe inflammation or associated with hydroxychloroquine therapy at the beginning of the treatment.

## 5. Conclusion

There is delayed diagnosis in all cases of DLE, ranging from 1 month to 25 years especially when the manifestation of the disease is a blepharitis. Distinguishing blepharitis associated with DLE can be difficult and initial misdiagnosis is common. To increase the clinical ability among ophthalmologists to perform a correct diagnosis, the following list reports the main features of blepharitis associated with DLE which is provided.

1. Lower eyelids are the site most involved.
2. The eyelids have lesions that appear as reddish patches with slight infiltration and tender deformity of the ciliary border.
3. Other skin anomalies most commonly localized on the face are present.
4. Blepharitis is mainly bilateral, but often asymmetrical and in few cases also monolateral.
5. There is failure to standard blepharitis treatment.
6. There is long history of blepharitis.
7. Blepharitis associated to DLE is most common in female gender and during the middle age.

Although ocular complications in CLE are quite rare, collaboration between rheumatologists, dermatologists, and ophthalmologists is fundamental to timely treat and prevent further complications. Ophthalmologists should be trained to detect these anomalies and refer to the specialist in doubtful cases.

## Figures and Tables

**Table 1:** Clinical features of ocular DLE in each study.

Reference/country	Study design	Age (years)	Ratio F : M	Ratio Monolateral : Bilateral	Ocular manifestation	Ratio ANA P : N	Treatment	Duration before diagnosis
Feiler Ofry et al. (1979), Israel [17]	CR	17	1 : 0	0 : 1	Blepharoconjunctivitis	—	Hydroxychloroquine 500 mg/day	48

Huey et al. (1983), USA [18]	CS	28.2 ± 5.5	6 : 1	0 : 7	Blepharoconjunctivitis	—	—	48.2 ± 29.9

Donzis et al. (1984), USA [19]	CR	39.5 ± 3.5	1 : 1	2 : 0	Periorbital edema; lid lesions	0 : 1	Hydroxychloroquine^*∗*^	7 ± 7

Tosti et al. (1987), Italy [20]	CS	53.3 ± 8.5	2 : 1	2 : 1	Blepharitis	0 : 3	Chloroquine phosphate^*∗*^	36 ± 33.9

Ziv et al. (1986), Israel [21]	CR	36	1 : 0	0 : 1	Eyelid plaque	0 : 1	Hydroxychloroquine 500 mg/day; intralesional corticosteroid	84

Raizman and Baum (1989), USA [23]	CS	51 ± 16.9	1 : 1	2 : 0	Stromal keratitis	1 : 0	Prednisolone acetate 1% eye drops	1

Meiusi et al. (1991), USA [24]	CR	29	0 : 1	1 : 0	Lid lesion	—	—	48

Cyran et al. (1992), USA [26]	CS	48 ± 4.2	2 : 0	1 : 1	Periorbital edema and erythema	1 : 1	Quinacrine 100 mg/day; hydroxychloroquine 200 mg twice; prednisone 40 mg/day	62 ± 82

Bettis et al. (1993), USA [27]	CR	21	0 : 1	0 : 1	Blepharitis	1 : 0	—	—

Gloor et al. (1997), USA [28]	CS	42 ± 8.4	2.0	1 : 1	Blepharitis	1 : 0	Hydroxychloroquine^*∗*^	32 ± 36.7

Uy et al. (1999), USA [29]	CR	58	1 : 0	0 : 1	Hypertrophic conjunctival mass	—	Hydroxychloroquine^*∗*^	180

Williams and Ramos-Caro (1999), USA [30]	CR	36	1 : 0	0 : 1	Periorbital mucinosis	1 : 0	Prednisone 60 mg/day; hydroxychloroquine 250 mg twice a day	12

Akagi et al. (1999), Japan [31]	CS	61 ± 2.8	2.0	0 : 2	Plaque periocular regions	1 : 0	No treatment/spontaneous resolution	9 ± 4.2

Gasior-Chrzan and Ingvarsson (1999), Norway [32]	CR	66.5 ± 7.7	2 : 0	1 : 1	Blepharitis	0 : 2	Hydroxychloroquine 200 mg twice	42 ± 25.4

Galeone et al. (2014), Italy [33]	CR	33	1 : 0	1 : 0	Blepharitis; lid lesion	1 : 0	Hydroxychloroquine^*∗*^	36

Thorne et al. (2002), USA [34]	CS	51.5 ± 34.6	2 : 0	0 : 2	Blepharitis; symblepharon	2 : 0	Hydroxychloroquine; prednisolone 50 mg a day	31.5 ± 40.3

Acharya et al. (2005), USA [35]	CS	43 ± 12.27	4 : 1	5 : 0	Chronic blepharoconjunctivitis	0 : 5	Hydroxychloroquine^*∗*^	106 ± 129.59

Giménez-García et al. (2005), Spain [36]	CR	23	0 : 1	0 : 1	Blepharitis	0 : 1	Hydroxychloroquine 250 mg/day	—

Ena et al. (2005), Italy [37]	CR	25	1 : 0	0 : 1	Blepharitis	—	Hydroxychloroquine^*∗*^	24

Au (2006), UK [38]	CR	39	0 : 1	1 : 0	Blepharitis	—	Topical corticosteroid	4

Pandhi et al. (2006), India [39]	CS	47.5 ± 3.5	1 : 1	0 : 2	Blepharoconjunctivitis	1 : 1	Hydroxychloroquine 500 mg/day	—

Koga et al. (2006), Japan [40]	CR	39	1 : 0	1 : 0	Lid erosive erythema	0 : 1	Prednisolone 10 mg/day	144

Gunasekera et al. (2008), UK [41]	CR	24	1 : 0	1 : 0	Lid lesion	—	Hydroxychloroquine 200 mg twice	2

Ricotti et al. (2008), USA [42]	CR	38	1 : 0	1 : 0	Lid lesion and edema	0 : 1	Mycophenolate mofetil 1 g twice daily and hydroxychloroquine^*∗*^	60

Vukicevici and Milobratovic (2010), Serbia [43]	CR	56	1 : 0	1 : 0	Blepharoconjunctivitis	1 : 0	Hydroxychloroquine 500 mg/day	—

Yaghoobi et al. (2010), Iran [44]	CR	28	1 : 0	0 : 1	Blepharitis	—	Hydroxychloroquine^*∗*^	24

Papalas et al. (2011), USA [45]	CS	52.1 ± 17.1	7 : 1	7 : 1	Eyelid lesions and plaques	—	CO_2_ laser; topical corticosteroid and ointment	26.3 ± 21.2

Serarslan et al. (2011), Turkey [46]	CR	33	1 : 0	1 : 0	Periorbital edema and erythema	0 : 1	Hydroxychloroquine 200 mg twice	24

Gupta et al. (2012), UK [47]	CS	46.8 ± 14	6 : 1	4 : 3	Periorbital swelling; blepharitis	1.4	Hydroxychloroquine^*∗*^; spontaneous resolution; intralesional corticosteroid	37.57 ± 28.78

Erras et al. (2012), Morocco [48]	CR	26	1 : 0	0 : 1	Eyelid swelling	0 : 1	Hydroxychloroquine^*∗*^	—

Ghauri et al. (2012), UK [49]	CS	43.5 ± 9.7	4 : 0	4 : 0	Blepharitis; lid lesion	—	Hydroxychloroquine^*∗*^	68.25 ± 67.2

Kopsachilis et al. (2013), Greece [50]	CR	45	1 : 0	0 : 1	Blepharitis	—	Hydroxychloroquine 200 mg twice	144

Arrico et al. (2014), Italy [51]	CR	37	1 : 0	1 : 0	Proptosis and orbital myositis	1 : 0	Methylprednisolone (1 g/day) for 3 days followed by oral prednisolone 20 mg/day	—

Kono et al. (2014), Japan [52]	CR	42	1 : 0	0 : 1	Orbital myositis	1 : 0	Methylprednisolone 1 g/day for three days followed by oral prednisolone 20 mg/day	120

CR: case report; CS: case series; F: female; M: male; P: positive; N: negative.

^*∗*^Dosage not specified.

**Table 2:** Clinical features of ocular LEP in each study.

Reference/country	Study design	Age (years)/gender	Ratio Monolateral : Bilateral	Ocular site	Ratio ANA P : N	Treatment
Sheehan-Dare and Cunliffe (1988), UK [22]	CR	47 m	Monolateral	Periorbital edema	Positive; speckled pattern	Prednisolone 40 mg

Magee et al. (1991), USA [25]	CR	41 m	Monolateral	Periorbital swelling and proptosis	—	Hydroxychloroquine and prednisone^*∗*^

Inuzuka et al. (2001), Japan [56]	CR	71 f	Bilateral	Eyelid plaque and subcutaneous nodules	Positive	Prednisolone 30 mg

Kao et al. (2010), USA [55]	CR	76 m	Monolateral	Enophthalmos	Positive; diffuse pattern	Prednisone 60 mg

Sudhakar et al. (2012), USA [54]	CR	51 f	Monolateral	Orbit and periorbit inflammation associated with CRAO	—	—

Ohsie et al. (2012), USA [53]	CR	18 f	Monolateral	Panniculitis involving orbit and periorbit tissue	Negative	Corticosteroid and mycophenolate mofetil^*∗*^

CR: case report; P: positive; N: negative; LEP: lupus erythematosus profundus; CRAO: central retinal artery occlusion.

^*∗*^Dosage not specified.
